# Established and emerging prognostic factors in mycosis fungoides and Sézary syndrome

**DOI:** 10.3389/fonc.2026.1881519

**Published:** 2026-07-01

**Authors:** Viviane Liao, Robert N. Stuver, Shamir Geller

**Affiliations:** 1 Dermatology Service, Department of Medicine, Memorial Sloan Kettering Cancer Center, New York, NY, United States; 2 Lymphoma Service, Department of Medicine, Memorial Sloan Kettering Cancer Center, New York, NY, United States; 3 Department of Dermatology, Weill Cornell Medicine, New York, NY, United States

**Keywords:** cutaneous T-cell lymphoma, individualized, mycosis fungoides, prognostic factors, Sézary syndrome

## Abstract

Mycosis fungoides (MF) and Sézary syndrome (SS) are the most common subtypes of cutaneous T-cell lymphoma (CTCL), characterized by heterogeneous clinical behavior and variable prognosis. Accurate prognostication is essential for risk-adapted management. This review synthesizes emerging evidence on prognostic factors in MF and SS, with a focus on recent genomic advances. Traditional prognostic frameworks are outlined, highlighting the prognostic impact of demographics, stage, clinical features, and histologic findings. More advanced prognostics are then outlined, including genomic alterations and impact of the tumor microenvironment. We highlight realms in which integration of traditional and more biological prognostic frameworks can support more individualized treatment strategies.

## 1 Introduction

Cutaneous T-cell lymphomas (CTCLs) are a heterogeneous group of T‐cell non-Hodgkin lymphomas primarily involving the skin (1–3). Mycosis fungoides (MF) is the most common subtype, caused by the malignant transformation of skin-resident effector memory T cells and defined by a generally indolent clinical course, whereas Sézary syndrome (SS) is a rare, leukemic variant derived from thymic memory T cells, generally with a more aggressive course with significant blood involvement and erythroderma (1, 2, 4). In the vast majority of patients, neither MF nor SS is immediately life-threatening, and therefore for most patients the primary goals of therapy are to optimize symptom control, minimize treatment-related side effects, and control or minimize disease burden (3, 5–7). Understanding disease- and patient-related prognostic factors that may predict disease progression and be associated with long-term outcomes is an integral component in the ongoing management of patients with MF/SS. In this narrative review, we focus on the evolving landscape of prognostic factors, highlighting established paradigms and more recent advances, without undertaking a formal systematic search strategy or risk-of-bias assessment.

## 2 Conventional prognostic schemas and factors

### 2.1 TNMB classification and clinical stage

Traditional Lugano staging is not used in MF/SS, which is instead dictated by the International Society for Cutaneous Lymphomas (ISCL), United States Cutaneous Lymphoma Consortium (USCLC), and European Organisation for the Research and Treatment of Cancer (EORTC) guidelines (3). The original tumor-node-metastasis-blood (TNMB) staging of MF/SS was developed over 40 years ago and has undergone several revisions, most recently in 2022, with an emphasis on blood staging and preferred methods, the use of clonality for various disease compartments, and clarification of visceral staging (3). The TNMB staging translates into a clinical stage (IA through IVB). In clinical practice, full TNMB staging is challenging, in particular in regard to lymph node classification, which requires biopsy and use of a formal lymph node classification system (see the National Cancer Institute-Veterans Administration [NCI-VA] or Dutch Criteria), bone marrow biopsies, which are not routinely performed for early-stage or even advanced-stage disease, and clonality assessment, which requires clonal sequencing across the skin, nodes, viscera and blood.

Nevertheless, stage (or at least an estimation of stage) remains the most readily available and established prognostic factor in MF/SS (8). Several large, historical observational studies demonstrate the importance of stage and prognosis. For example, in a series of over 1000 patients from the MD Anderson Cancer Center in Houston, TX, United States, treated between 1982 and 2009, progression-free survival (PFS), disease-specific survival (DSS), and overall survival (OS) were significantly different across TNMB staging, T1–T4 classification, and B0–B2 staging (9). These findings have been corroborated in other large series such as that from the Stanford Cutaneous Lymphoma Program and a United Kingdom cohort which validated the ISCL/EORTC staging criteria (10, 11). Specifically, patients with limited skin involvement, such as T1 classification or clinical stage IA, have long survival that matches control populations (10). In general, higher TNMB classification and/or clinical stage is associated with reduced survival (**Table 1**); however, some studies suggested that some cases with T4 (erythrodermic disease, stage III) may actually have slightly better survival than T3 (tumor stage disease, stage IIB) (15, 16).

**Table 1:** MF/SS survival outcomes by stage across series.

Reference	Agar 2010 (11)	Talpur 2012 (9)	Miyashiro 2022 (12)	Chen 2023 (13)	Scarisbrick 2025 (14)
Patient number	1502 all-stage	1263 all-stage	727 all-stage	461 all-stage	552 advanced-stage
Cohort nationality	United Kingdom	United States	Brazil	China	International
Time frame	1980-2009	1982-2009	1989-2018	2009-2021	2015-2025
OS by clinical stage	5-year OS, % IA: 94 IB: 84 IIA: 78 IIB: 47 IIIA: 47 IIIB: 40 IVA1: 37 IVA2: 18 IVB: 18	N/A	5-year OS, % IA: 99.1 IB: 98.5 IIA: 74.8 IIB: 76.5 IIIA: 60.0 IIIB: 77.1 IVA1: 46.2 IVA2: 36.8 IVB: 33.5	5-year OS, % IA: 95.7 IB: 93.2 IIA: 95.7 IIB: 70.1 IIIA: 55.5 IIIB: 50.0 IVA1: 25.0 IVA2: 22.5 IVB: 25.0	5-year OS, % IIB: 50.0 IIIA: 64.8 IIIB: 43.9 IVA1: 50.8 IVA2: 25.9 IVB: 36.9
OS by T stage	5-year OS, % T1a: 97 T1b: 91 T2a: 85 T2b: 81 T3: 44 T4: 41 T4 (3): 20	Median OS, years T1: NR T2: 26.26 T3: 5.96 T4: 5.00	5-year OS, % T1a: 100 T1b: 91.3 T2a: 100 T2b: 84.6 T3: 74.8 T4: 56.7 T4 (3): 38.1	OS, adjusted HR T1: 1 T2: 1.03 T3: 6.43 T4: 15.6	Death rate per 100 person-years T1: 10.7 T2: 11.7 T3: 15.7 T4: 13.9
OS by N stage	5-year OS, % N0: 83 N1: 54 N1a: NR N1b: 57 N2: 25 N2b: NR N3: 19 Nx: 40	Median OS, years N0: 26.26 N1: 16.91 N2: 5.43 N3: 6.13 N4: 2.85	N/A	OS, adjusted HR N0: 1 N1: 2.68 N2: 14.29 N3: 23.86 Nx: 0	Death rate per 100 person-years N0: 10.9 N1: 8.8 N2: 11.3 N3: 25.2 Nx: 20.2
OS by M stage	5-year OS, % M0: 75 M1: 17	Median OS, years M0: N/A M1: 4.42	N/A	OS, adjusted HR M0: 1 M1: 11.31	N/A
OS by B stage	5-year OS, % B0: 76 B0a: 87 B0b: 63 B1: 34 B2: 26	Median OS, years B0: 29.28 B1: NR B2: 4.64	N/A	OS, adjusted HR B0: 1 B1: 6.88 B2: 37.58	Death rate per 100 person-years B0: 14.5 B1: 13.2 B2: 14.4 Bx: 13.8

CI, confidence interval; HR, hazard ratio; N/A, not applicable; NR, not reached; OS, overall survival.

It is important to note that, essentially, all long-term data on stage and prognosis emanates from before the widespread use of more targeted systemic therapies, such as denileukin diftitox (17), romidepsin (18), brentuximab vedotin (19, 20), mogamulizumab (21), pembrolizumab (22), E7777 (23), and lacutamab (24). While some recent reports suggest improved DSS in the past decade (25), the long-term impact of these therapies on prognosis remains to be fully determined, particularly for those with early-stage disease who experience stage progression and for those with advanced-stage disease.

### 2.2 Blood involvement

Blood stage (B) is the most recently added prognostic factor to the TNMB staging system and deserves specific attention (26). Blood stage currently has three classifications based on disease burden in absolute number, with further sub-categorization based on whether circulating disease is clonal and identical to skin-based disease (7). This is a change from the original classification, which used percentages of circulating cells in addition to absolute number. While the prognostic value of blood disease burden is well established, this change in definition alters blood stage for some patients and may affect interpretation of prognosis. Specifically, a 2024 study from Northwestern University, Evanston, IL, United States, comparing patients’ staging based on either the 2007 or 2022 ISCL/EORTC guidelines found that some patients (11%) who later developed leukemic transformation were reclassified from B2 to B1 under the updated criteria (27). This phenomenon occurs due to the shift from percentage-based thresholds to absolute counts, which may underestimate disease burden in lymphopenic patients (27). The authors of this report proposed refining the 2022 guidelines to re-incorporate percentage-based measures alongside absolute counts to improve prognostic accuracy (27).

It is a challenge to understand whether B1 disease is associated with stage migration or survival, given shifts in definition as described above, as well as lack of uniform assessment for circulating disease in early-stage skin disease. Additionally, there is lack of consensus among guidelines regarding the use of flow cytometry for the assessment of blood tumor burden. The recommendation of measuring blood tumor burden by flow cytometry was included in the latest EORTC guidelines update from 2023 (7). Most studies on the impact of blood burden in early-stage disease are, therefore, heterogenous and challenging to interpret. Nevertheless, some studies suggest that low-level, monoclonal blood involvement is a risk factor for disease progression and is associated with suboptimal response (28–32). For example, one study of 388 patients with early-stage MF found that low-level blood involvement at diagnosis was associated with increased risk of progression to advanced-stage disease (28). Furthermore, the Cutaneous Lymphoma International Prognostic Index (CLIPi) study developed two prognostic indices for early (IA–IIA) and late stage (IIB–IVB) disease based on multivariate data from 1,502 patients with MF/SS. The study identified B1/B2 as an adverse prognostic factor, with ten-year OS of 53.2% for low-risk versus 15.0% for high-risk patients (29).

Beyond its role in the formal TNMB staging system, where, in erythrodermic disease, B1 blood involvement upstages patients from IIIA to IIIB (31), the presence of B1 has several practical management implications. In our practice, we generally do not factor B1 disease into clinical decision-making for patients with stage IA or IB disease. However, in certain clinical scenarios, such as intractable disease or refractory MF/SS-associated itch, the presence of B1 may trigger earlier consideration of systemic therapy, particularly ECP or mogamulizumab (31–33). There are currently no prospective data that addresses whether B1 in early-stage disease carries sufficient prognostic implications to justify earlier initiation of systemic therapy or whether it warrants closer surveillance.

Importantly, clonality status may be more prognostically meaningful than the quantitative B-class alone, as reported by the landmark validation study by Agar et al. of 1,502 patients with MF/SS. In this study, occurrence of B0b (clone-positive blood without meeting the B1 threshold) was an independent predictor of poor survival and disease progression in a multivariate analysis of clinical factors associated with outcomes (11). Marks et al. similarly found that concordant positivity for both TCR gene rearrangement and flow cytometry was associated with inferior survival in patients with early-stage CTCL (30). These findings underscore the complexity of interpreting blood involvement and clonality, resulting in the lack of consensus among guidelines regarding the use of flow cytometry blood assessment.

### 2.3 Histological subtype and large cell transformation

Several variants of MF have been described, namely folliculotropic MF (FMF), pagetoid reticulosis, and granulomatous slack skin (1). Compared to the latter two, which are indolent, FMF has been associated with a risk of stage progression (1, 34, 35), akin to that of tumor-stage MF (34, 36). There is some suggestion that FMF could be classified into two variants: an advanced-stage, which exhibits the most widely recognized clinicopathological manifestations of FMF and has an aggressive course, and an early-stage, exhibiting distinct clinicopathological features with a more favorable prognosis (37, 38). FMF has a deeper localization of the neoplastic infiltrate and may be less accessible to skin-targeted therapies, though this finding in and of itself does not markedly influence therapeutic decision-making. Large cell transformation (LCT), defined as large cells present in >25% of lymphoid/tumor cell infiltrates in a skin lesion biopsy, is often a clinically aggressive feature with therapeutic implications (39). In multiple prognostic models, the presence of LCT is an independent prognostic factor associated with reduced survival (14, 40). If present, CD30 in LCT represents a therapeutic target with brentuximab vedotin (39, 41).

Other than CD30, histochemical markers that have been studied for prognostic assessment in MF include Ki-67, GATA3, p53, immune checkpoint molecules, and signal transducer and activator of transcription (STAT3) activation (**Table 2**). High CD30 expression is linked to LCT, higher stage, and decreased PFS and OS, although specifically in transformed MF evidence suggests that high CD30 expression may actually reduce risk of death (42–44). Elevated Ki-67 and GATA3 levels are associated with worse OS and PFS, with Ki-67 levels also correlating with higher disease stage (13, 43, 45, 46, 48, 50). p53 overexpression is often associated with LCT (47, 49), although limited data are available on its association with survival outcomes in MF. STAT3 correlates with advanced stage and LCT, but not consistently with survival (48, 51). Similarly, programmed cell death protein 1 (PD-1) shows variable prognostic significance, although a high combined expression score of inducible T-cell co-stimulator (ICOS), programmed death-ligand 1 (PD-L1), and PD-1 correlates with advanced disease, LCT, and decreased OS (52). These immunohistochemical markers provide additional insight into disease biology and risk stratification in MF and may enhance prognostic accuracy beyond clinical staging alone.

**Table 2:** Prognostic immunohistochemical markers.

IHC marker	Molecule type and function	Early-stage/non- transformed MF	Transformed MF/LCT	Advanced MF/SS	Survival outcomes (OS/PFS/DSS)
CD30	Molecular type: Transmembrane receptor Function: Promotes cell survival through TNF-receptor associated proteins (42)	High dermal expression: increased stage at diagnosis, increased max stage, and decreased OS (43)	High expression associated with decreased hazard of death (42)	High expression associated with LCT, increased risk of progression, and decreased PFS (44)	Decreased PFS and OS (in LCT and some non- transformed MF) (42–44)
Ki-67	Molecular type: Cell-cycle marker Function: Promotes cell proliferation (43)	Higher expression: increased stage at diagnosis, higher clinical stage, shorter OS (43)	-	-	Ki-67 >60%: decreased OS, DSS, PFS (45); Ki-67 >30% in LN biopsy: decreased OS (13)
GATA3	Molecular marker: Transcription factor Function: Regulates T-cell development and function (46)	-	High expression: decreased OS (47)	-	GATA3 ≥60%: decreased PFS and OS (46, 48)
p53	Molecular marker: Tumor suppressor Function: Modulates cell cycle and apoptosis in response to DNA damage (49)	-	p53 >30% more prevalent in transformed MF/SS vs. non-transformed MF (49)	Associated with LCT (48)	Linked to adverse outcomes in transformed disease and LCT (47, 49)
STAT3	Molecule type: Transcription factor Function: Promotes cell proliferation, immune suppression, and angiogenesis (48)	-	High GATA3 with low STAT3/T-bet: decreased OS (50)	High expression: associated with advanced MF (51)	High expression not significantly associated with OS or PFS alone (51)
Immune checkpoint markers (PD-1/PD-L1/ICOS)	Molecule type: Immune checkpoints Function: Downregulates host antitumor immune response (52)	-	-	High ICOS or PD-L1: associated with advanced-stage MF/SS and LCT (51); PD-1: not significantly associated with disease stage or LCT (52)	High combined checkpoint score (PD-1, PD-L1, ICOS): associated with advanced -stage disease, LCT, and decreased OS (52); PD1 >25% in LN biopsy decreased OD (45)

ICOS, inducible T-cell co-stimulator; IHC, immunohistochemical; LCT, large cell transformation; LN, lymph node; MF, mycosis fungoides; OS, overall survival; PD-1, programmed cell death protein 1; PD-L1, programmed death ligand 1; PFS, progression-free survival; SS, Sézary syndrome; TNF, tumor necrosis factor.

It is important to note that essentially all long-term survival data presented in this review and in **Tables 1**-**3** were generated prior to the widespread use of more targeted systemic therapies, such as brentuximab vedotin, mogamulizumab, or pembrolizumab (53–55). While some recent reports suggest improved survival in the past decade (20–22, 56), the long-term impact of these therapies on overall survival has not yet been determined. Given the above and the chronic nature of MF/SS, it will likely require several additional years of prospective follow-up before their effect on survival and disease progression can be reliably assessed. As such, the survival figures in **Table 1**, while representing the best available evidence, may not fully reflect outcomes achievable under current treatment paradigms, and this represents an important limitation of all existing prognostic studies.

**Table 3:** Established prognostic indices in MF/SS.

Prognostic index	CLIPI (Benton 2013) (29)		CLIC (Scarisbrick 2015) (16)	PROCLIPI (Scarisbrick 2025) (14)
Number of patients	1502 all-stage		1275 advanced-stage	552 advanced-stage
Time frame	1982–2009		2007–2015	2015–2025
Prognostic factors for worse survival identified	Early-stage model: • Age >60 years • Male sex • Presence of plaques • Folliculotropic disease • N1/Nx status Late-stage model: • Age >60 years • Male sex • N2/N3 status • B1/B2 stage • M1		• Stage IV • Age >60 years • Elevated serum LDH • LCT	• N3 status • Age >60 years • Elevated serum LDH • LCT in skin
Risk groups and OS	Early-stage model (10-year OS, %)	Late-stage model (10-year OS, %)	5-year OS, %	5-year OS, %
Low (0 or 1 factors)	90.3	53.2	67.8	63.3
Intermediate (2 factors)	76.2	19.8	43.5	44.7
High (3+ factors)	48.9	15.0	27.6	18.3

CLIC, Cutaneous Lymphoma International Consortium; CLIPI, Cutaneous Lymphoma International Prognostic Index; LCT, large cell transformation; LDH, lactate dehydrogenase; MF, mycosis fungoides; OS, overall survival; PROCLIPI, Prospective Cutaneous Lymphoma International Prognostic Index; SS, Sézary syndrome.

### 2.4 Prognostic indices

Various prognostic indices incorporating stage with other patient- and disease-related factors can delineate certain patients with low-risk and high-risk disease (**Table 3**). In the aforementioned validation study of the ISCL/EORTC staging system using a cohort of over 1500 patients treated at a multidisciplinary cutaneous lymphoma clinic in the United Kingdom, multivariate analyses identified T classification, B0b blood classification (as compared to B0a), FMF, LCT, serum lactate dehydrogenase (LDH), and tumor distribution as independent risk factors for stage progression (11). As for DSS and OS, TNMB classification, age, sex, LDH, and various clinicopathological features (FMF, LCT, and tumor distribution) were all independent risk factors (11). Findings from this cohort were validated against the previously mentioned MD Anderson cohort to develop the CLIPi, which delineates low-, intermediate-, and high-risk groups for early- and late-stage disease based on the presence of various adverse factors (29). Soon after this study, the Cutaneous Lymphoma International Consortium (CLIC) used a cohort of almost 1,300 patients to develop a similar low-, intermediate-, and high-risk prognostic model using four risk factors (stage IV, age >60 years, elevated LDH, and LCT) (16). More recently, a large, international effort (Prospective Cutaneous Lymphoma International Prognostic Index, PROCLIPI) reported a revised CLIPI, again using four independent risk factors (N3, age >60 years, elevated LDH, and LCT) to delineate low-, intermediate- and high-risk groups (14). Each of these scoring systems provides additional information to consider when making treatment decisions, but they do not markedly influence treatment decisions over a pure stage-based approach currently.

### 2.5 Demographics: race

Black patients with MF have significantly worse OS across multiple reports, even when accounting for prognostic factors such as stage, age, and sex, and when controling for socioeconomic factors (57–61). A matched case–control study of 292 patients from our center showed that Black patients were diagnosed approximately 9 years younger, were predominantly female, and had worse overall survival (HR 2.88, 95% CI 1.21–6.85; p=0.017) (59). Another study, the largest multicenter cohort to date, comprising 883 Black patients with CTCL across seven institutions, confirmed younger age at presentation, more advanced stage at diagnosis, and reduced survival in Black patients aged 60 years or older, with more than double the rate of *Staphylococcus aureus* bacteremia compared to White patients (62, 63). Furthermore, analyses of 9,351 patients with MF from the SEER and National Cancer Database similarly identified African American race as an independent predictor of inferior OS (57, 58). The mechanism underlying these prognostic disparities remains unclear. At least one study has shown a higher occurrence of LCT among Black patients (61). Our institutional data identified morphologic features that correlate with disease progression and survival: hypopigmented MF confers a favorable prognosis, with hypopigmentation being protective in Black patients (HR 0.04, 95% CI 0.01–0.33; p=0.002), whereas erythroderma and ulceration carry disproportionately worse survival risk in Black compared to White patients (64).

Importantly, race is a social construct and an imperfect proxy for biologic variation. To address this, our group is conducting an ongoing study using genetic ancestry analysis inferred from tumor sequencing data (MSK-IMPACT) rather than self-reported race. Preliminary results from 161 patients demonstrate that patients of African genetic ancestry were diagnosed 10 years younger than those of European/Ashkenazi Jewish ancestry (p=0.01) and were less likely to present with stage IA disease (0% vs. 23.2%; p=0.01), with trends toward higher disease-related mortality and worse PFS. These findings confirmed known racial disparities through a genomic framework and supported the need for ancestry-informed research to disentangle biologic from socioeconomic contributors to the observed prognostic differences in MF/SS (65).

## 3 Emerging prognostic factors

Among the emerging prognostic factors discussed in this review, important distinctions should be made regarding their current level of evidence. The strongest support exists for TCR clonality assessment in the blood and its concordance with skin-based clones, which has been associated with disease progression and suboptimal treatment response across multiple independent studies and is referenced in the 2022 ISCL/EORTC staging update and the 2023 EORTC treatment guidelines (3, 7), although it is not uniformly performed in clinical practice due to variability in standardization and availability. At an intermediate level, targeted genomic profiling has identified recurrent mutations and copy-number alterations that correlate with aggressive clinical behavior and reduced OS in multiple retrospective cohorts as reported in **Table 4**. These studies are limited by small sample sizes, an inherent challenge given the rarity and clinical heterogeneity of MF/SS, and lack of prospective validation. At the most exploratory level, single-cell and spatial transcriptomic signatures, JAK2 fusions, tumor microenvironment gene expression scores and compositional analyses, remain confined to small proof-of-concept studies and currently serve primarily to advance biological understanding rather than clinical prognostication. Importantly, beyond the question of evidence, the advanced molecular techniques required for many of these emerging markers remain largely inaccessible to many centers worldwide, limiting their near-term clinical applicability even as evidence matures. None of these emerging markers have been prospectively validated or incorporated into clinical guidelines, and their potential to independently improve risk stratification beyond conventional staging and validated prognostic indices remain to be determined through collaborative, prospective efforts.

**Table 4:** Select studies on genomic/transcriptomic factors in MF/SS.

Study	Patients included	Genetic methods used	Select genomic findings
da Silva Almeida, 2015 (66)	25 SS, 8 MF, 9 other CTCL	WES	Non-leukemic MF: • Low CNA burden Disease progression: Not evaluated SS: • Deletions in tumor suppressors (*TP53, RB1, PTEN, DNMT3A, CDKN1B*) • Mutations in epigenetic modulators (*TET2, CREBBP, MLL3, BRD9, SMARCA4, CHD3*) • Gains in chromosome 7, 8q, and 17q Survival: Not evaluated
Argyropoulos, 2020 (67)	21 early MF, 15 advanced MF/LCT, 17 SS, 12 CD30^+^ LPD, 5 gdTCL, 7 other rare CTCLs	Targeted 585 gene panel	Early-stage disease: • Low mutational burden Disease progression: Not evaluated Advanced-stage disease: • High mutational burden • Mutations in *CDKN2A/B, PCLO*, *FAT1*, or *TP53* Survival: • Mutation in at least one of *CDKN2A/B, PCLO, FAT1, TP53* among non early-stage MF CTCLs correlates with aggressive immunopathological features, increased tumor burden, and decreased OS
Park, 2021 (68)	94 CTCL all-stage, and previously published DNA-seq data from 203 patients	WGS, WES, RNA-seq	Early-stage disease: • Fewer PD-1 deletions Disease progression: Not evaluated Advanced-stage disease: • Structural variants (*17pdel, 10qdel, 17qamp*) among leukemic CTCLs • Deletions in *TP53, NFKB2, ARID1A, MGMT, GRAP, AGAP6* among leukemic CTCLs • Mutations in tumor suppressors (*ARID1A, CDKN2A, ZEB1*) among leukemic CTCLs • PD-1 deletion Survival: • PD-1 deletion predicts shorter survival, higher peak tumor-cell burden, and higher LDH in stage-matched patients
Rindler, 2021 (69)	3 patients with advanced-stage MF	scRNA-seq on patches versus plaques/tumors	Early-stage disease: • Higher expression of *CD69, HSPA1A, ZFP36* in patches Disease progression: • Downregulation of *CXCR4, CD69, HSPA1A, ZFP36, TXNIP, IL7R* among malignant clones in plaque/tumor lesions • Downregulation of SELL-CD34, CXCR4-CXCL12 and CCR4-CCL5 malignant clone and non-malignant cell interactions in plaque/tumor lesions Advanced-stage disease: • Higher expression of *CCR7, SELL, CD27, IGFL2, KIR3DL2, IL16, IKZF2* in patches Survival: Not evaluated
Fléchon, 2024 (70)	48 patients with all-stage MF	WES	Early-stage disease: • del17q11.2 (*SUZ12, NF1*) Disease progression: • Acquisition of *JUNB*, gain10p15.1 (*IL2RA/IL15RA*), del12p13.1 (*CDKN1B*), or del6q16.3 (TNFAIP3) Advanced-stage disease: • High mutational burden • gain7q, gain10p15.1 (*IL2RA*/*IL15RA*), del10q24.32 (*NFKB2*), del10p11.22 (*ZEB1*), mutations in *JUNB* and *TET2* Survival: • del10p11.22 (*ZEB1*), gain10p15.1 (*IL2RA/IL15RA*), gain7q, and del6q16.3 (*TNFAIP3*) associated with shorter OS

CNA, copy number alteration; CTCL, cutaneous T-cell lymphoma; DNA-seq, DNA sequencing; gdTCL, gamma-delta T-cell lymphoma; LDH, lactate dehydrogenase; MF, mycosis fungoides; OS, overall survival; RNA-seq, RNA sequencing; scRNA-seq, single-cell RNA sequencing; SS, Sézary syndrome; WES, whole exome sequencing; WGS, whole genome sequencing.

### 3.1 Genomic profiling

Recent advancements in genomic profiling have uncovered numerous genetic alterations with potential prognostic relevance in MF/SS. These include mutations, deletions, and copy number variations affecting genes involved in cell-cycle regulation, epigenetic modification, differentiation, proliferation, and immune checkpoint expression (52, 66, 68, 70–73) (**Table 4**).

MF is characterized by recurrent mutations in *JUNB*, *TET2*, *MAPK1*, *FOXA1*, *FLT4*, and *PLCG1*, as well as alterations in the Janus kinase (JAK)/STAT pathway, including *STAT3*, *STAT5A*, *STAT5B*, and *JAK3* (70). Additional mutations affect tumor-suppressor and signaling genes such as *TP53*, *TMEM259*, *SUZ12*, *NF1*, *NOTCH1*, *CARD11*, *ZEB1*, *TNFAIP3*, *CDKN2A*, and *CDKN2B*, *FAT1*, and *KMT2D* (70, 73). Epigenetic regulators, including *SMARCA4*, *MLL2*, *MLL3*, and *SETBP1* may also be altered (66, 73). SS demonstrates a distinct genetic profile with prevalent deletions involving tumor suppressors such as *TP53*, *RB1*, *PTEN*, *DNMT3A*, and *CDKN1B*, as well as the *MYC* oncogene (66, 72). Other implicated genes in SS include those involved in epigenetic regulation (*TET2*, *CREBBP*, *MLL2*, *MLL3*, *BRD9*, *SMARCA4*, *CHD3*, *ARID1A*, *CTCF*) and signaling (*MAPK1*, *BRAF*, *CARD11*, *PRKG1*, *STAT5B*, *ZEB1*, *NFKB2*) (72, 74). Importantly, while these recurring aberrancies are observed, MF/SS has a complex mutational landscape, with high diversity among patients and even within the same patient upon clonal evolution, highlighting the diverse repertoire of T-cell biology in general and the challenges inherent in relying solely upon mutation data in prognostication. However, data from our center showed that mutations in at least one of four recurrently mutated genes, *CDKN2A/B*, *PCLO*, *FAT1*, and *TP53*, were associated with higher mutational burden, aggressive immunopathological features, and decreased OS in patients with non-early MF forms of CTCL, identifying these alterations as potential surrogate markers for aggressive disease (67).

The data on gene fusions and prognosis in MF reveals a complex landscape. Fusion transcripts identified in tumor-stage MF include *DOT1L, KDM6A, LIFR, TP53*, and *TP63* fusions, though their individual prognostic significance has not been established (75). However, *JAK2* fusions were recently reported in patients with MF, and were predominantly found in patients with indolent disease courses, mainly early-stage MF (75), therefore it is important to further assess genetic fusions for their prognostic significance.

Beyond evaluating recurrent mutations, additional work has surveyed mutation burden, how certain pathways may drive disease phenotype, and dynamic changes over time that occur in those with stage progression. Work from our center has shown that advanced-stage MF has the highest mutational burden of all CTCL subtypes, whereas early-stage MF has the lowest, with one-third of cases harboring no detectable genomic alterations (67). This is consistent with other studies demonstrating that non-leukemic, early-stage MF has significantly fewer copy number alterations than SS and fewer structural copy number variations than leukemic MF and SS (66, 68). MF cases with LCT also exhibit a higher overall mutation rate than non-transformed MF, with activating oncogenic *RAS* mutation identified exclusively in LCT cases, suggesting a potential role for these alterations in driving LCT (76).

Similarly, integrated genomic studies have demonstrated diverse genomic pathways that may drive the equally diverse clinical presentations of MF/SS. For example, deletions in six genes (*TP53*, *NFKB2*, *ARID1A*, *MGMT*, *GRAP*, and *AGAP6*) are more frequent in leukemic MF and SS compared to skin-limited MF, likely reflecting their localization within large, recurrent deletions characteristic of leukemic CTCL (68). Subsequent studies have shown that distinct chromosomal and mutational events, including del10p11.22 (*ZEB1*), gain10p15.1 (*IL2RA* and *IL15RA*), gain7q, and del6q16.3 (*TNFAIP3*), may be associated with disease progression and shortened OS (70). Longitudinal clonal evolution analyses have further demonstrated that progression may be accompanied by acquisition of *JUNB* mutations, gain of 10p15.1 (*IL2RA*/*IL15RA*), or del12p13.1 (*CDKN1B*) (70). Similarly, interrogation of lesions at varying time points over the disease course have associated progression with downregulation of the tissue residency markers *CXCR4* and *CD69*, the heat shock protein *HSPA1A*, the tumor suppressors and immunoregulatory mediators *ZFP36* and *TXNIP*, and *IL7R* within the malignant clone. Moreover, these genes are upregulated in malignant cells residing in clinically unaffected skin, indicating a switch in molecular phenotype during disease progression (69). Indeed, patches from patients with advanced-stage MF (in comparison to patches from patients with early-stage MF) show upregulation of *CCR7*, *SELL*, *CD27*, *IGFL2*, *KIR3DL2*, *IL16*, and *IKZF2*, indicating that these transcriptional changes may serve as markers of advanced disease at the patch stage (69). A recent study also identified a molecular signature predictive of MF progression, developed by detecting genes that were continuously upregulated throughout progression from patch- to plaque- to tumor-stage MF. A high progression signature was associated with stage progression, radiation therapy, and a low treatment response rate among patients with stage I MF (69).

MicroRNAs (miRNAs) are small non-coding RNAs that regulate post-transcriptional protein expression and have been increasingly implicated in the prognosis of MF. Several studies have demonstrated differential miRNA expression between early- and advanced-stage MF, including miRNAs encoded by the oncogenic miR-17/92, 106b/25, and 106a/363 clusters (77). Compared with patients with non-progressive disease, patients with progressive MF exhibited upregulation of multiple miRNAs, such as miR-155, miR-21, let-7i, miR16, miR-142-3p, miR-146b-5p, miR-92a, miR-92, and miR-106a, which have previously been implicated in advanced-stage disease (77). Building upon these early investigations into the role of miRNA in MF progression, a three-miRNA classifier, based on miR-106b-5p, miR-148a-3p, and miR-338-3p, was shown to stratify patients with early-stage MF into high- and low-risk groups for disease progression, with significantly different PFS (78). This classifier outperformed established prognostic factors, including sex, age, patch/plaque T stage, and the CLIPI score, in predicting progression within a cohort of 154 patients with early-stage MF (78).

T-cell receptor (TCR) clonotype patterns have also been investigated using TCR next-generation sequencing, revealing associations between specific clonotypes and disease severity. In patients with early-stage MF, a tumor clone frequency of >25% in the skin was a stronger predictor of disease progression than any other established prognostic factor (79). The clonal TCR Vb20 segment is associated with advanced-stage disease as well as folliculotropism with LCT (80). Similarly, the Vg8 clonotype was more frequently observed in advanced-stage than early-stage MF and was associated with poorer OS (80). These findings highlight the potential prognostic utility of TCRβ and TCRγ clonotypes and underscore the value of TCR sequencing (80).

### 3.2 Tumor microenvironment

**Figure 1:**
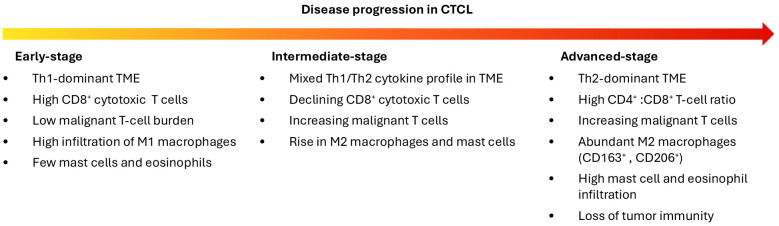
Changes in the tumor microenvironment with increasing disease stage. CTCL, cutaneous T-cell lymphoma; Th, T-helper; TME, tumor microenvironment.

The tumor microenvironment (TME) plays a critical role in the malignant transformation and persistence of MF/SS. Malignant T cells manipulate the inflammatory environment to facilitate their expansion by suppressing cellular immunity and anti-tumor responses while fostering chronic inflammation (81–83). Early-stage CTCL is generally characterized by a T helper (Th) 1-dominant TME, with enrichment of reactive CD4^+^ Th1 cells and CD8^+^ cytotoxic T cells involved in the cell-mediated antitumor response (82, 83). In contrast, disease progression generally involves a shift towards Th2 polarization and a decline in Th1-associated cytokines and chemokines, a transition thought to facilitate immune evasion in CTCL (82–84) (**Figure 1**).

The TME is also characterized by expression of PD-1 and its ligands in tumor cells and cells of the microenvironment (84–86). Patients with advanced-stage MF/SS generally display higher immune checkpoint-related gene expression (*PD-1*, *PD-L1*, *LAG3*, *TIM3*, *CTLA4*, *ICOS*, *FOXP3*, *IL10*) than early-stage patients or healthy controls (88). More recent studies have demonstrated that higher expression of *PD-L1* is associated with advanced-stage MF and LCT (52). Moreover, *PD-1* deletions, which may reverse T-cell exhaustion due to loss of *PD-1* expression (68), not only increase with advancing stage, but are also associated with higher tumor burden, elevated LDH, and shorter survival versus stage-matched controls (68).

Tumor-associated macrophages are also known to play a role in maintaining the TME in MF. Upregulation of M2 macrophages in plaque-stage compared to patch-stage MF highlights the role of pro-tumorigenic M2 polarization in disease progression (89). While CD206 and CD163 are both common markers of M2 polarization, selective increase in CD206^+^ but not CD163^+^ macrophages with tumor progression in MF suggests enrichment of a Th2-driven macrophage phenotype, consistent with known inflammatory changes in advanced-stage MF 87).

Mast cells have also been shown to play a pro-tumorigenic role in CTCL, contributing to induction of cytokine production and malignant T-cell proliferation. CTCL lesions demonstrate increased mast cell infiltration and degranulation, most prominently at the lesion periphery (90). Furthermore, higher mast cell counts are found in patients with progressive disease compared to those with non-progressive disease (90). Finally, tissue eosinophil infiltration may carry prognostic significance. In early-stage MF, increased tissue eosinophil abundance has been associated with worsened OS and PFS, as well as higher likelihood of subsequent lymph node involvement (91).

Cancer-associated fibroblasts (CAFs), which are enriched in MF lesions, are also thought to promote malignant T-cell proliferation and contribute to a Th2-polarized TME (89, 92). MF tumor cells may drive CAF generation through SOX4 and TGF-β-mediated reprogramming of normal fibroblasts, while CAFs in turn increase invasiveness and metastatic potential of malignant T cells via the interleukin (IL)-6/JAK2/STAT3/SOX4 and IL-6/HIF-1α/SOX4 pathways (92). Within this positive feedback loop, SOX4 plays a critical regulatory role, with greater SOX4 expression correlating with shorter OS and PFS (92). Consistent with the proposed role of CAFs in MF progression, immunohistochemical (IHC) staining for CAF markers increases with advancing tumor stage, with higher staining intensity in progressive stage I MF than indolent stage I MF (89). TIMP1, an inducer of CAF accumulation, is likewise significantly upregulated during disease progression and associated with higher likelihood of progression in patients with Stage I MF, further supporting a role for CAF expansion in MF progression (89).

In addition to stromal components, tumor-infiltrating B-cell lymphocytes have emerged as important contributors to MF pathogenesis and progression. Lesional CTCL skin demonstrates increased B-cell abundance compared to healthy controls and other inflammatory dermatoses (89, 93). Early transcriptomic studies reported significantly higher expression of MS4A1 (CD20) in advanced-stage than early-stage MF, suggesting a link between B-cell infiltration and disease severity (94). More recently, single-cell and spatial transcriptomic analyses have demonstrated that B cells within lesional MF skin are organized into tertiary lymphoid structures (93), and increased B-cell abundance has been shown to correlate with disease progression and worsened PFS (93–95).

To facilitate clinical application of TME-related events, a nine-gene TME gene signature, including *CCL13*, *CCL23*, *CCL26*, *CCL18*, *TRAP*, *FCER2*, *CCL24*, *CD209*, and *LILRB3*, has been described (96). Routine application of this signature is not performed, though it highlights the promise of TME interrogation and prognostication.

## 4 Limitations

While this narrative review provides a comprehensive synthesis of the current literature, the lack of a formal systematic search strategy means certain relevant studies may have been inadvertently omitted, limiting the transparency and reproducibility of the selection process. Furthermore, because a structured risk-of-bias assessment was not undertaken, the analysis focuses on summarizing the overarching themes rather than objectively grading the methodological quality of the individual evidence. Acknowledging these scoping boundaries, the insights presented offer a valuable thematic framework while highlighting areas that merit more rigid methodological evaluation in future research. Additionally, it is important to acknowledge that the long-term survival data and associated tables presented in this review primarily reflect historical outcomes established prior to the widespread clinical adoption of modern targeted systemic therapies, such as brentuximab vedotin, mogamulizumab, and pembrolizumab. Because MF and SS are chronic in nature, the definitive impact of these contemporary interventions on survival outcomes requires several more years of prospective follow-up to be fully quantified. Consequently, while the data included represents the most comprehensive evidence currently available, they may not entirely capture the improved survival outcomes achievable under today’s evolving treatment paradigms.

## 5 Conclusion

Due to the clinical and biologic heterogeneity of MF/SS, management is complex and driven by multiple patient- and disease-related factors. Disease prognostication is helpful in such management. Despite immense and ever-increasing genomic studies, use of molecular features in prognostication is not routine and has lagged behind biological findings. In practice, clinical factors, in particular stage, still dominate prognostic attempts and therapeutic decision-making. However, as highlighted in this review, recent dedicated attempts to understand the biological factors driving disease presentation, stage migration, and clonal evolution shed light upon the heterogeneity of MF/SS. Advancements in the understanding of prognostic factors in CTCL has been very helpful for refining treatment selection based on their individual patient characteristics and will continue to improve over the next decade as new data are collected.
